# Zinc Improves Functional Recovery by Regulating the Secretion of Granulocyte Colony Stimulating Factor From Microglia/Macrophages After Spinal Cord Injury

**DOI:** 10.3389/fnmol.2019.00018

**Published:** 2019-02-01

**Authors:** Xian Li, Shurui Chen, Liang Mao, Daoyong Li, Chang Xu, He Tian, Xifan Mei

**Affiliations:** ^1^Department of Orthopedics, The First Affiliated Hospital of Jinzhou Medical University, Jinzhou, China; ^2^Department of Oncology, The First Affiliated Hospital of Jinzhou Medical University, Jinzhou, China; ^3^Department of Histology and Embryology, Jinzhou Medical University, Jinzhou, China

**Keywords:** zinc, spinal cord injury, microglia/macrophages, NF-kappa B, G-CSF

## Abstract

While zinc promotes motor function recovery after spinal cord injury (SCI), the precise mechanisms involved are not fully understood. The present study aimed to elucidate the effects of zinc and granulocyte colony stimulating factor (G-CSF) on neuronal recovery after SCI. The SCI model was established by Allen’s method. Injured animals were given glucose and zinc gluconate (ZnG; 30 mg/kg) for the first time at 2 h after injury, the same dose was given for 3 days. A cytokine antibody array was used to screen changes in inflammation at the site of SCI lesion. Immunofluorescence was used to detect the distribution of cytokines. Magnetic beads were also used to isolate cells from the site of SCI lesion. We then investigated the effect of Zinc on apoptosis after SCI by Transferase UTP Nick End Labeling (TUNEL) staining and Western Blotting. Basso Mouse Scale (BMS) scores and immunofluorescence were employed to investigate neuronal apoptosis and functional recovery. We found that the administration of zinc significantly increased the expression of 19 cytokines in the SCI lesion. Of these, G-CSF was shown to be the most elevated cytokine and was secreted by microglia/macrophages (M/Ms) *via* the nuclear factor-kappa B (NF-κB) signaling pathway after SCI. Increased levels of G-CSF at the SCI lesion reduced the level of neuronal apoptosis after SCI, thus promoting functional recovery. Collectively, our results indicate that the administration of zinc increases the expression of G-CSF secreted by M/Ms, which then leads to reduced levels of neuronal apoptosis after SCI.

## Introduction

Spinal cord injury (SCI) is one of the most common central nervous system injuries encountered in clinic and is often accompanied with the loss of motor and sensory function. Consequently, SCI can exert serious effects on human health and quality of life (Sekhon and Fehlings, 2001; Schwab et al., 2018). Unfortunately, there are no efficient treatment options with which to handle this fatal condition.

Zinc is an essential trace element necessary for the survival of many cell types. Many enzymes and transcription factors exert their active function by creating covalent bonds with zinc ions (Kaur et al., 2014). According to Andreini, there are approximately 2,800 proteins in the human body that can potentially bind to zinc (Andreini et al., 2006). For example, zinc is present throughout the central nervous system and plays a crucial role in synaptic transmission and neuroregulation, and is thought to be involved in neuroprotection (Ripps and Chappell, 2014). Many studies have shown that zinc is involved in neural diseases including depression, Parkinson’s disease, Alzheimer’s disease and lateral sclerosis of the spinal cord (Yasui et al., 1993; Nowak et al., 2005; Brewer et al., 2010). However, our own previous data demonstrated that zinc promotes SCI recovery by up-regulating zinc transporter protein-1 and promoting the synthesis and release of brain-derived neurotrophic factors (Wang et al., 2011a). Nevertheless, zinc alters the disease process after SCI in many aspects. Following SCI, inflammation is the most overwhelming process and causes significant pathological changes, including apoptosis (Yeiser et al., 2002; Seth et al., 2015; Tian et al., 2018).

In order to evaluate the effect of zinc during the acute stages of SCI, we used a cytokine antibody array to detect the inflammatory changes involved contusion. As expected, after zinc treatment, many cytokines showed changes, and many cells showed alterations in functional activity. We also found that zinc promotes SCI recovery not only by elevating brain-derived neurotrophic factor but also by promoting microglia/macrophages (M/Ms) secreting the granulocyte colony stimulating factor (G-CSF) which protect the survival of neuron form the secondary injuries.

## Materials and Methods

### Establishment of the SCI Model and Drug Treatment

This study was carried out in accordance with the recommendations of the Guidelines for the Care and Use of Laboratory Animals published by the US National Institutes of Health. The protocol was approved by the Animal Care and Use Committee of Liaoning Medical University. Adult C57BL/6J mice (A total of 182 mice, 91 males and 91 females, 22–25 g) were purchased from Beijing Vital River Laboratory Animal Technology Co., Limited in Beijing, China, and housed in standard cages (five per cage) in a specific pathogen-free laboratory animal center in Jinzhou Medical University. Mice were maintained at 22 ± 1°C on a 12–12 h light-dark cycle and were acclimatized to their environment for 1 week prior to experimentation with free access to food and water. To induce SCI, we anesthetized all animals with 10% (w/v) chloral hydrate (0.33 ml/kg, i.p.). Then, we performed a laminectomy at the T9 vertebrae after first exposing the vertebral column. The spinal cord was fully exposed, then moderate contusive injury was conducted by striking with an impactor (2 mm diameter, 12.5 g, 5 cm in height) at the surface of the spinal cord at T9. Sham group mice underwent the same surgical procedure but were not subjected to contusion Postoperatively, urinary bladders were emptied twice daily. After 2 h, half of the mice were injected with ZnG (30 mg/kg i.p.; Biotopped, Beijing, China) and given daily 30 mg/kg doses until the third day. The rest of the mice were injected with an equivalent dose of vehicle (isosmotic glucose).

### Cytokine Antibody Array

The RayBio^®^ C-Series Mouse Cytokine Antibody Array C2000 (Raybiotech, Atlanta, GA, USA) was used to detect cytokine changes in the spinal cord after ZnG or vehicle treatment (*n* = 3). During SCI, cytokines mainly exist in the extracellular fluid. In order to extract cytokine proteins, we injected Brefeldin A to inhibit the secretion of cytokines in mice 6 h before taking samples. Thereafter, we can indirectly detect changes of cytokines by determining the levels of intracellular cytokines. We then extracted protein from the spinal cord tissue (1.5 cm in length). Extracts were first quantified with bicinchoninic acid Protein Assay Kit (P0010, Beyotime, Beijing, China). Then, the extract was diluted to 5 mg/ml with blocking buffer, and 100 μl of the protein sample was extracted for further use in this experiment. The cytokine assay was set up in accordance with the manufacturer’s instructions. Each antibody array (printed side facing up, Supplementary Figure S1) were placed into a well of the incubation tray, and incubated for 30 min with 2 ml blocking buffer at room temperature. Then, 100 μl of the protein sample was diluted to 1 ml, added into the hole on the array and incubated overnight at 4°C. After washing, 1 ml of biotinylated antibody cocktail was absorbed into each hole and incubated at 4°C overnight. After a further washing step, 2 ml of Horseradish Peroxidase-streptavidin was added into each hole and incubated overnight at 4°C. After consecutive washes, we then added 500 μl of the detection buffer mixture onto each membrane and incubated these for 2 min at room temperature. Last, we transferred the membranes to a CCD camera and exposed them. The intensity of the positive control signal (biotin) and negative control signal [phosphate-buffered solution (PBS)] was used to normalize the cytokine signal between the two arrays.

### Western Blot (WB) Analysis

Spinal cord tissues (1.5 cm length from the injury epicenter) and cells were collected for protein assay. The tissues and cells were homogenized in RIPA lysis buffer containing PMSF buffer (P0013B, Beyotime, Beijing, China) for 30 min on ice. After centrifugation at 12,000 RMP (25 min, 4°C) to remove debris, the supernatant was quickly stored at −80°C. Extracts were first quantified with bicinchoninic acid Protein Assay Kit (P0010, Beyotime, Beijing, China). Then, tissue samples containing 40 μg of protein were separated by sodium salt-polyacrylamide gel electrophoresis (SDS-PAGE) before being transferred to polyvinylidene fluoride (PVDF) membranes and incubated with the appropriate primary antibodies overnight, after which they were incubated with horseradish peroxidase-conjugated secondary antibodies for 2 h. Finally, bands were detected by BeyoECL Plus (Beyotime, Beijing, China), and signals visualized by a Tanon 5500 Gel Imaging System (Tanon, Shanghai, China).

### Quantitative Real-Time PCR Analysis (qRT-PCR)

After the mice were killed by excessive anesthetic, a 1.5 cm length of spinal cord tissue was taken from the injured point for experiment of quantitative real-time PCR (qRT-PCR), or all M/Ms in the 1.5 cm length of spinal cord tissue were isolated by immunomagnetic cell separation techniques for experiment of qRT-PCR. Total RNA extracts were obtained using TRIzol Reagent (Ambion, Foster City, CA, USA), and 5 μg of total RNA was used to synthesize cDNA (promega, Fitchburg, WI, USA). qRT-PCR was performed using SYBR Green (Promega, Fitchburg, WI, USA). cDNA samples were amplified on a 7500 fast RT-PCR system (Applied Biosystems) under the following conditions: 95°C for 3 min, followed by 40 cycles of 95°C for 15 s and 60°C for 45 s. The relative expression levels of the target genes were normalized to those of the housekeeping gene ribosomal protein S18 (RPS18) and the target genes from the experimental group were compared with the corresponding target genes from the control group using the (1 + e)^−ΔΔCT^ method (Heid et al., 1996). The primers for G-CSF and RPS18 are given in Table 1.

**Table 1:** Sequences for mouse quantitative real-time PCR (qRT-PCR) primers.

Primer name	Forward sequence	Reverse sequence
RPS18	AGGATGTGAAGGATGGGAAG	TTCTTCAGCCTCTCCAGGTC
HPRT1	AGTGTTGGATACAGGCCAGAC	CGTGATTCAAATCCCTGAAGT
G-CSF	GCTGGAAGGCAGAAGTGAAGG	TGCAGGCTCTATCGGGTATTT

### Immunofluorescence Staining

*In vivo* experiment involved the cardiac perfusion of SCI mice with 0.9% sodium chloride, experiments concluded with 4% paraformaldehyde. The spinal cord tissue was then removed and placed in 30% sucrose for 3 days. After that, samples were cut into 10 μm sections horizontally, transversely or sagittally, and the slides were kept in a cryoprotective solution at −80°C.

First, frozen slices were placed at room temperature for 2 h, then rinsed three times with PBS. Sections were then incubated for 15 min at room temperature in 0.3% Triton X-100 and then incubated for 2 h with 5% goat serum. Then, sections were incubated in primary antibody at 4°C overnight in a damp box. Primary antibodies were as follows: anti-G-CSF (1:750, Abcam, Cambridge, UK); anti-NeuN (1:1,000, Abcam, Cambridge, UK); anti-GFAP (1:450, Dako, Denmark); anti-Iba-1 (1:1,000, Abcam, Cambridge, UK); anti-CC-1 (5 μg/ml, Merck, CA, USA); anti-cleaved-caspase3 (1:400, CST, Waltham, MA, USA), anti-Bcl-2 (1:1,000, Abcam, Cambridge, England) and anti-Bax (1:1,000, Abcam, Cambridge, England). Sections were then washed with PBS three times (5 min each) and then incubated with Alexa Fluor 488/568 FITC rabbit-anti-mouse secondary antibody (1:400, invitrogen, Carlsbad, CA, USA) for 2 h at room temperature. Nuclei were counterstained by 4′,6-diamidino-2-phenylindole (DAPI; Invitrogen, Carlsbad, CA, USA) for 15 min. Sections were then imaged with a fluorescent microscope (Olympus, Tokyo, Japan) using equal exposure times. The ratio of the number of positive cells relative to the total number of cells in the same multiple field of vision was then calculated.

### Immunomagnetic Cell Separation and Flow Cytometry

M/Ms were isolated from SCI lesions. In brief, mice from the SCI-Vehicle and SCI-ZnG groups (*n* = 6 per group) were sacrificed 3 days after injury. Tissue samples were then acquired by dissecting out 2 cm long pieces of spinal cord using the lesion site as a center; these samples were then placed on ice. The following isolation experiment was then conducted according to the manufacturers kit guidelines for the isolation and cultivation of microglia from adult mouse or rat brain (Miltenyi Biotec, Bergisch Gladbach, Germany). In brief, we first prepared enzyme 1 mix and enzyme 2 mix, and then placed the spinal cord tissues and enzyme 1 mix and enzyme 2 mix together in tube C, which was closed tightly and attached onto a gentle MACS Octo dissociator (Miltenyi Biotec, Bergisch Gladbach, Germany). We then ran the gentle MACS program 37C_ABDK_01 of the gentle MACS Octo dissociator. The sample was then resuspended and applied onto a 70 μm strainer in order to collect the flow through. Debris and red blood cells were then removed by density gradient centrifugation and erythrocyte lysis. Then, the cell suspension was incubated with rat CD11b/c microglia microbeads for 15 min in the dark at 4°C. Finally, the cell suspension was applied onto a LS column, and the labeled cells collected magnetically on the LS column. For purity analysis, we resuspended up to 10^6^ cells per 49.375 μL of buffer, and added 0.625 μL of APC anti-mouse/human CD11b antibody (Biolegend, San Diego, CA, USA). The resulting sample was mixed well and incubated for 10 min in the dark at 4°C. After separation, cells labeled with microbeads were used as an isolated sample; using a pre-separated sample as a control. Cell purity was analyzed on a FACSCalibur flow cytometer (BD Biosciences, San Jose, CA, USA) using FlowJo Software (Tree Star, Inc., Ashland, OR, USA).

### Cell Culture

RAW 264.7 cells were obtained from the Chinese Academy of Sciences Cell Bank and grown at 37°C in a humidified atmosphere containing 5% CO_2_ using RPMI 1640 medium (Gibco, Grand Island, NY, USA) supplemented with 10% of fetal bovine serum, 100 units/ml of penicillin, 100 μg/ml of streptomycin (Gibco, Grand Island, NY, USA).

### Cell Viability Measurements

Cell viability was assessed using a CellTiter 96^®^ Aqueous One Solution Cell Proliferation Assay (MTS; Promega, Fitchburg, WI, USA) assay in accordance with the manufacturer’s protocol. In brief, RAW264.7 cells were seeded into 96-well plates (5,000 cells/well) and incubated for 24 h. Then, different doses of ZnG (0, 50, 60, 70, 80, 90, 100, 200, 400, 600, 800 and 2,000 μM) were added to the wells for 12 h and 24 h. After treatment, 20 μl of MTS was added to the wells, and the plates were cultured for 2 h. Then, absorbance was measured at 490 nm using a microplate reader (Varioskan Flash, Thermo scientific, Waltham, MA, USA).

### Nissl Staining

Sections of spinal cord were incubated in 0.1% Nissl staining solution (Beyotime, Beijing, China) for 3 min at 37°C, rinsed with distilled water, dehydrated in 95% and 100% ethanol solutions for 2 min and then cleared in xylene for 5 min. In each section, Nissl-stained images were then captured on both sides of the ventral horn under an optical microscope (DMI4000B, Leica, Wetzlar, Germany).

### Transferase UTP Nick End Labeling (TUNEL) Staining

Sections obtained 3 days after SCI were used for Transferase UTP Nick End Labeling (TUNEL) staining. First, we placed frozen slices at room temperature for 2 h to thaw. Sections were then rinsed three times with PBS. Sections were then fixed in freshly-prepared 4% paraformaldehyde in PBS (pH 7.4) for 20 min at room temperature. The tissues were then washed for 30 min with PBS. Slides were then incubated in freshly-prepared permeabilization solution (0.1% Triton X-100, 0.1% sodium citrate) for 10 min at 4°C. The slides were then rinsed twice with PBS, and the TUNEL reaction mixture was added onto each section. Slides were then incubated in a humidified atmosphere for 60 min at 37°C in the dark. Slides were then rinsed three times in PBS and then incubated with DAPI for 15 min. Images were then captured using a fluorescence microscope (Olympus, Tokyo, Japan). Red TUNEL dots located within a blue-stained nucleus were defined as apoptotic cells. The total number of cells, and apoptotic cells, of three sections from each mouse were counted and then ratios were calculated.

### Quantitative Image Analysis

In order to quantify the number of viable spinal motoneurons in each group on the 21st day after SCI, transected spinal cord sections (four slices per animal) were selected, with the injury point as the center, from the ab initio (+3, +6 mm) to the cauda (−3, −6 mm). Neurons with a clear nucleolus and Nissl bodies were counted as described in previous literature (Li et al., 1994, 2017). According to Rexed’s lamina system of gray, the mean number of motor neurons in the bilateral ventral horn of the spinal cord (lamina IX) was manually calculated using ImageJ2x software (National Institute of Health, New York, NY, USA). In order to determine the ratio of apoptotic cells in the ventral horn of the spinal cord from each group, TUNEL staining was used as a test method; Red TUNEL dots located within a blue nucleus were identified as apoptotic cells. We selected two cross sections (two sections per animal) at two points (+4, −4 mm). The total number of cells, and apoptotic cells, in the three sections from each mouse were then counted and ratios calculated. In order to determine the number of apoptotic motor neurons in the ventral horn of the spinal cords from each group, we used the NeuN/cleaved-caspase-3/DAPI double staining method (Bai et al., 2017). NeuN/cleaved-caspase-3/DAPI double-positive cells were selected as representing positive SCI-induced apoptotic cells. For each animal, we selected two cross sections at two points (+4, −4 mm). The optical density of double-labeled cleaved caspase 3-positive neurons in the ventral motor regions was then quantified using ImageJ2X.

### Statistical Analysis

Data were analyzed using SPSS Software, version 19.0 (Chicago, IL, USA) and expressed as mean ± standard deviation (SD). Two experimental groups were analyzed using Mann-Whitney *U* test. In cases where there were more than two groups, we used one-way analysis of variance (ANOVA) followed by Bonferroni’s *post hoc* test when the variance was equal, and a Kruskal-Wallis test when the variance was uneven. Basso Mouse Scale (BMS) scores were analyzed by repeated measures two-way ANOVA, followed by Tukey’s *post hoc* test to compare the differences between each group on the same day. A *P* < 0.05 was considered to be statistically significant.

## Results

### Zinc Increases the Secretion of G-CSF in Spinal Cord Tissue After SCI

**Figure 1:**
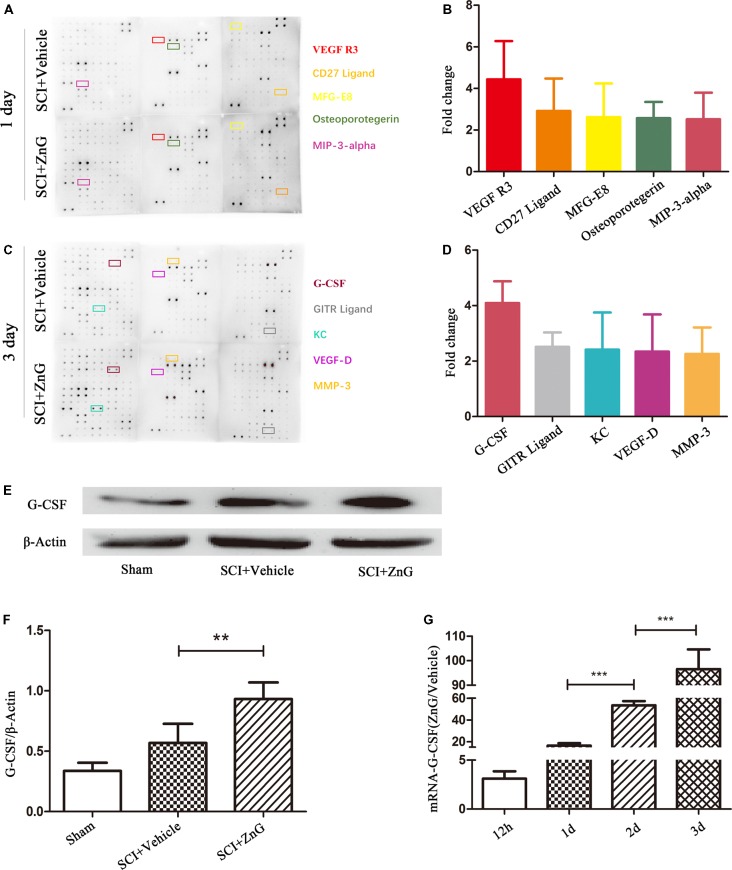
Granulocyte colony stimulating factor (G-CSF) was the highest elevated factor among the increasing cytokine profile in spinal cord tissue of acute spinal cord injury (SCI) mice after zinc treatment. The doublet spots in the membranes indicate each cytokine. **(A)** Intracellular proteins from spinal cord tissue were detected by a cytokine antibody array 1 day after glucose and ZnG treatment. **(B)** Densitometric analysis of (**A**; *n* = 3 per group). **(C)** The cytokine antibody array was repeated 3 days after glucose and ZnG treatment. **(D)** Densitometric analysis of (**C**; *n* = 3 per group). The expression of G-CSF in the spinal cord was verified by Western blotting (*n* = 6 per group, Mann-Whitney *U* test; **E,F**) and Quantitative real-time PCR (qRT-PCR; *n* = 6 per group, one-way analysis of variance (ANOVA) followed by Bonferroni’s *post hoc* test; **G**). Data are expressed as means ± standard deviation (SD). **p < 0.01, ***p < 0.001.

The pathological process of SCI involves the progression of inflammation (Anwar et al., 2016). We selected cytokine detection as the starting point with which to understand the pathological changes of SCI caused by zinc. The main change after SCI is a change in the levels of biologically-active cytokines (Mortazavi et al., 2015). We assessed the profiles of cytokine expression using a cytokine protein array using injured local spinal cord tissue from the SCI model treated with ZnG (*n* = 3) or vehicle (*n* = 3; Figures 1A–D). Then, we found that G-CSF in the zinc treatment group was higher on the first and third days than in the vehicle group. We then confirmed results arising from the cytokine protein array on the third day after injury, which showed that the expression of G-CSF in spinal cord after zinc treatment had increased in Western blotting (*P* < 0.01) and qRT-PCR (1 day vs. 2 days, 2 days vs. 3 days, *P* < 0.001; Figures 1E–G). Collectively, these results indicated that zinc increased the expression of G-CSF in spinal cord after SCI. Recent studies have reported that G-CSF acts as a neuroprotective and regenerative nutritional factor (Diederich et al., 2009; Henriques et al., 2010; Lee et al., 2012). Therefore, in studying the pathological effect of zinc in the treatment of SCI, G-CSF is a key cytokine for analysis. In our next experiment, aimed to identify the cells upon which zinc acts in order to increase levels of G-CSF.

### Elevated G-CSF Is Secreted by M/Ms After Zinc Treatment in SCI

**Figure 2:**
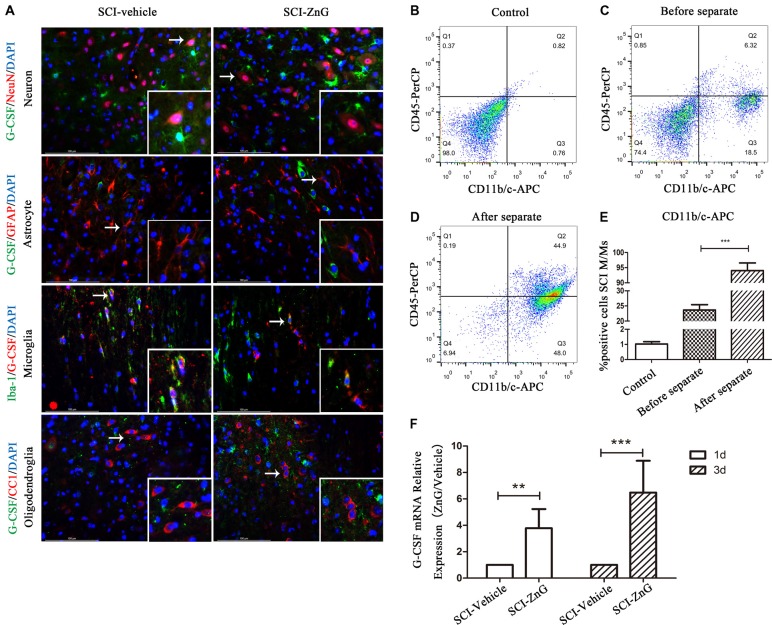
Elevated G-CSF was predominantly secreted by microglia/macrophages (M/Ms) after Zinc treatment during the acute stages of SCI. **(A)** The cellular localization of G-CSF was assessed by double immunofluorescent staining of G-CSF with NeuN, GFAP, Iba-1 and O4, respectively. Two transverse sections were taken from each animal at two points: +4 mm and −4 mm (*n* = 3 per group, white arrows: G-CSF-positive M/Ms). **(B–E)** The purity of M/Ms in each SCI lesion was determined by flow cytometry before and after isolation using the magnetic beads sorting method (*n* = 3 per group, Mann-Whitney *U* test). The purity of M/Ms was as high as 96% by sorting, meaning that such samples could serve in subsequent experiments. **(F)** The expression of G-CSF in M/Ms from each SCI lesion site was evaluated by qRT-PCR at the transcriptional level (*n* = 7 per group, Mann-Whitney *U* test). Data are expressed as means ± SD, **p < 0.01, ***p < 0.001.

To determine the expression profile and possible cellular source of G-CSF, we carried out immunofluorescent co-localization of G-CSF with neurons (NeuN), astrocytes (GFAP), M/Ms (Iba-1) and oligodendroglia (O4) in vehicle and ZnG treatment groups 3 days after SCI (Figure 2A). G-CSF was predominantly distributed in M/Ms after SCI mice were treated with zinc. In order to confirm the source of G-CSF, we also used immunomagnetic cell separation to obtain purified M/Ms from injured spinal cord; the purity of M/Ms in the cell population obtained by flow cytometry reached up to 96% (Figures 2B–E). Finally, we measured the mRNA expression of G-CSF in M/Ms by qRT-PCR on day 3 after zinc treatment (Figure 2F). Increased levels of G-CSF expression were detected in M/Ms on the third day after zinc treatment. These results were demonstrated in M/Ms at both the protein level by immunofluorescence co-localization and at the mRNA level. qRT-PCR results showed that the increased expression of G-CSF originated mostly from transcriptional activity in the M/Ms.

### Zinc Acts Directly on M/Ms to Increase G-CSF Expression

**Figure 3:**
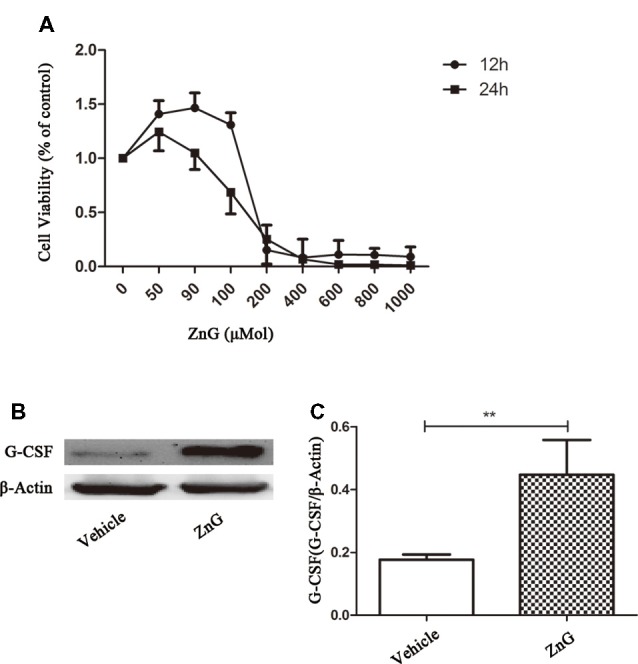
Zinc promoted M/M’s to directly express G-CSF. **(A)** The cellular viability of Raw264.7 cell was measured by MTT after exposure to different concentrations of ZnG (*n* = 4).** (B)** The expression of G-CSF by Western blotting. **(C)** Quantitative analysis of G-CSF expression. Data are expressed as means ± SD (*n* = 6 per group, Mann-Whitney *U* test). **p < 0.01.

The effect of zinc on the central nervous system can result in changes in the levels of many different types of enzymes and factors. To determine whether zinc plays a direct role in M/Ms, or whether zinc activates M/Ms indirectly, we studied the effects of zinc on M/Ms *in vitro* using RAW264.7 cells. The results of MTS zinc toxicity tests suggested that a ZnG concentration less than 90 μmol was non-toxic, and that cell viability was reduced to 25% when ZnG concentration was increased to 200 μmol over a 24 h period (Figure 3A). In the next experiment, we chose 90 μM as the processing condition and detected the expression of G-CSF at 24 h with or without zinc treatment. Our results showed that the expression level of G-CSF was higher than that of the control group (*P* < 0.01; Figures 3B,C). These results suggest that zinc acts directly on M/Ms and promotes the expression of G-CSF, rather than promoting M/Ms to express G-CSF *via* cytokines secreted by other cells.

### Zinc Can Increase the Expression of G-CSF by M/Ms *via* the NF-κB Signaling Pathway

**Figure 4:**
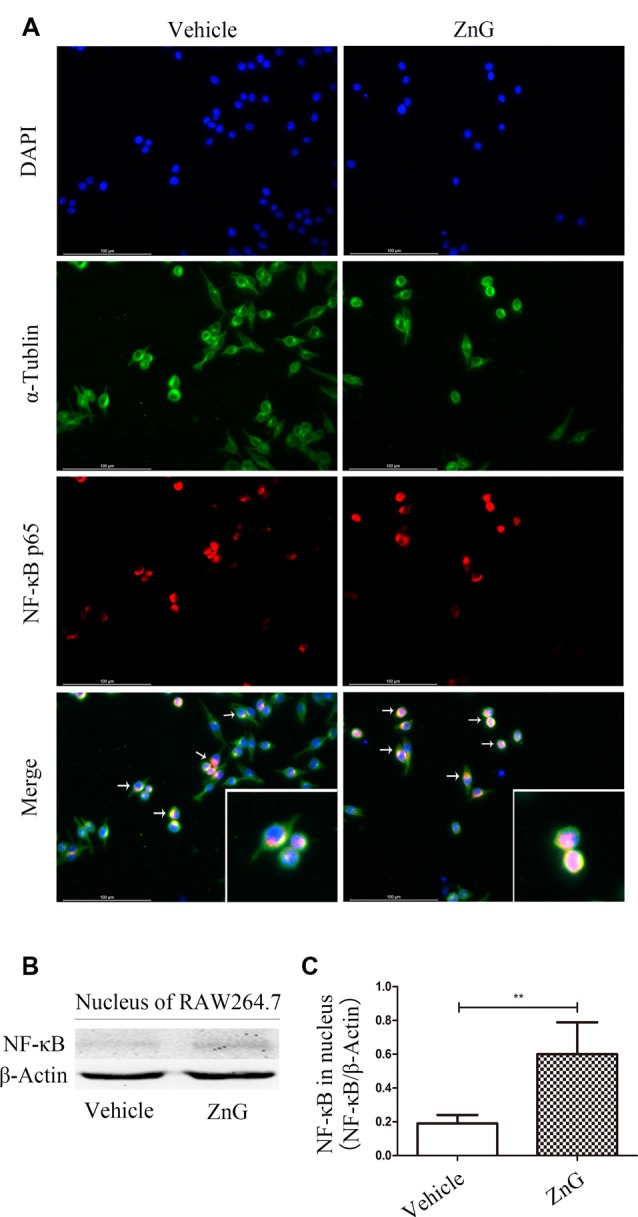
Zinc promoted the activation of nuclear factor-kappa B (NF-κB) and its entry into the nucleus. **(A)** Immunofluorescence staining demonstrated that NF-κB p65 underwent nuclear translocation upon ZnG stimulation in RAW264.7 cells. **(B)** The expression of G-CSF in the nucleus, as demonstrated by Western blotting. **(C)** Quantitative analysis of NF-κB expression in the nucleus of cells from the Vehicle and ZnG groups (*n* = 7 per group, Mann-Whitney *U* test). Data are expressed as means ± SD. **p < 0.01.

**Figure 5:**
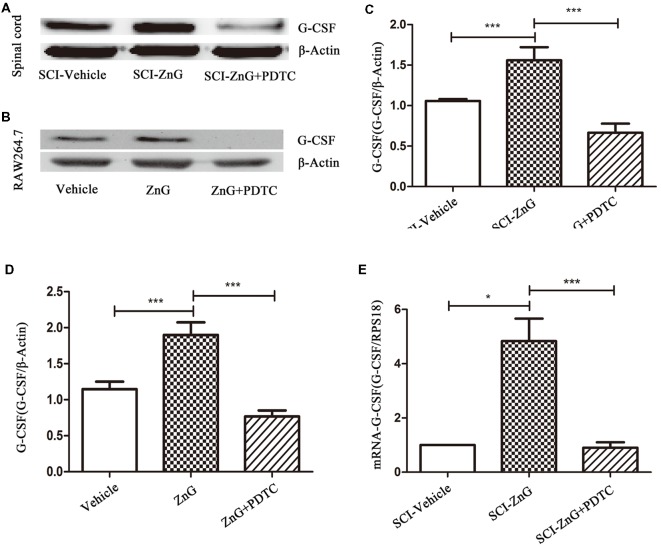
Zinc increased the expression of G-CSF *via* the NF-κB signaling pathway. Western blotting of indicated proteins at the SCI lesion site **(A,C)** or in Raw264.7 cells (**B,D**; *n* = 6 per group, one-way ANOVA followed by Bonferroni’s *post hoc* test). **(E)** Detection of the effect of NF-κB on G-CSF at the mRNA level in M/Ms after SCI, as determined by qRT-PCR. Data are expressed as means ± SD (*n* = 7 per group, Kruskal-Wallis test). *p < 0.05, ***p < 0.001.

Next, we attempted to identify whether the increase in G-CSF secretion by M/Ms in response to zinc treatment depended on the nuclear factor-kappa B (NF-κB) signaling pathway. NF-κB is located in the cytoplasm without stimulation signals, but in the presence of an external stimulus, the inhibition upon NF-κB is removed, and the activated NF-κB enters the nucleus to regulate the expression of related genes (Quivy and Van Lint, 2004). First, we used RAW264.7 cells to investigate whether zinc promoted the activation of NF-κB *in vitro*. Immunofluorescence results suggested that zinc can induce NF-κB to enter the nucleus (Figure 4A). In addition, we used a kit to extract nuclear protein, and then measured the amount of the nuclear fraction of NF-κB protein by Western blotting. Results showed that the expression of NF-κB in the nuclei of cells treated with ZnG was higher than that in the vehicle group (*P* < 0.001; Figures 4B,C). In order to further understand whether the activation of NF-κB plays a role in the zinc-induced expression of G-CSF, ammonium pyrrolidinedithiocarbamate (PDTC, 100 mg/kg *in vivo*, 20 μM *in vitro*), an inhibitor of the NF-κB signaling pathway, was given together with ZnG to block the activation of NF-κB. Western blotting analysis showed that the increased levels of G-CSF around the injury point after zinc treatment was significantly inhibited by PDTC *in vivo* (Figures 5A,C). The same results were evident in our *in vitro* experiments (Figures 5B,D). The expression of mRNA was detected by qRT-PCR in M/Ms isolated from the injured spinal cord (Figure 5E). We also found that the mRNA expression of G-CSF was significantly reduced after PDTC treatment, and that the level of expression was lower than that in the ZnG group (*P* < 0.001).

### Zinc Promote Histological Neural Survival and Functional Recovery by G-CSF After SCI

**Figure 6:**
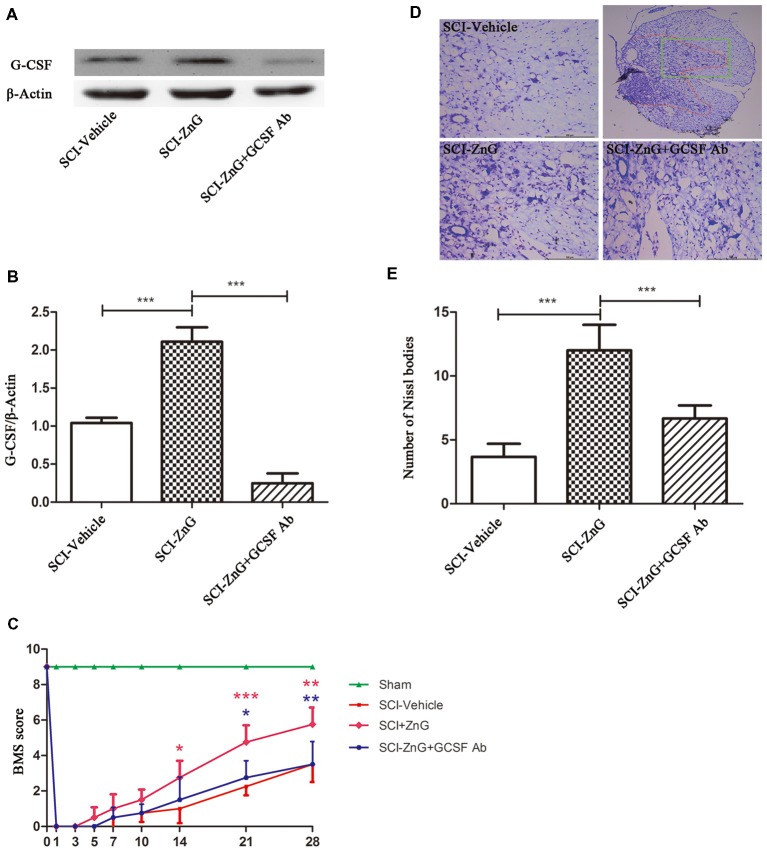
Zinc treatment promoted motor functional recovery by G-CSF following SCI. **(A)** The neutralizing effect of neutralizing antibody against G-CSF in SCI by Western blotting.** (B)** Quantitative analysis of G-CSF expression in different groups (*n* = 6 per group, one-way ANOVA followed by Bonferroni’s *post hoc* test). **(C)** Statistical analysis of motor functional recovery of Sham, SCI-Vehicle, SCI-ZnG and SCI-ZnG+GCSF Ab mice before and after SCI using Basso Mouse Scale (BMS) scores (*n* = 4–5 per group, two-way ANOVA followed by Tukey’s *post hoc* test). **(D)** Representative sections showing normal neurons that contained prominent nucleoli, loose chromatin, and Nissl bodies. **(E)** Quantitative analysis of motor neurons in the ventral horn at 21 days post-injury (*n* = 6 per group, one-way ANOVA followed by Bonferroni’s *post hoc* test). Data are expressed as means ± SD, *p < 0.05, **p < 0.01, ***p < 0.001.

Many research suggests that G-CSF exerts its anti-neuronal apoptosis effect, through which protect neuron from secondary injuries in rats brain ischemia-reperfusion injury (Henriques et al., 2010; Khorasanizadeh et al., 2017). So, we consider whether zinc plays a neuroprotective and anti-apoptotic role mainly through G-CSF. G-CSF neutralizing antibody were used to study the role of G-CSF in zin-induced spinal cord protection. Mice were injected intraperitoneally with G-CSF neutralizing antibody at a dose of 10 μg/day after SCI (Cedervall et al., 2015), for 3 days. Western blotting showed that G-CSF neutralizing antibody did play a role in neutralizing G-CSF (Figures 6A,B). At the same time, we also observed that zinc could promote the recovery of motor function after SCI, as evidenced by BMS scores, and that G-CSF played a role in this process (Figure 6C). We observed that the behavioral differences between the SCI-ZnG group and the SCI-Vehicle group began to be statistically significant on the 21st day after SCI (SCI-ZnG vs. SCI-Vehicle, 21 days, *P* < 0.001), and that the behavioral differences between the SCI-ZnG group and the SCI-ZnG+GCSF Ab group began to be statistically significant on the 21st day after SCI (SCI-ZnG vs. SCI-ZnG+GCSF Ab, 21 days, *P* < 0.05). Therefore, we carried out a follow-up experiment on day 21; Nissl staining showed that the number of motoneurons in the spinal ventral horn of mice treated with ZnG was higher than that of vehicle and ZnG+GCSF Ab mice (SCI-ZnG vs. SCI-Vehicle, *P* < 0.001; SCI-ZnG vs. SCI-ZnG+GCSF Ab, *P* < 0.001; Figures 6D,E). It was therefore evident that G-CSF plays an important role in the effects of zinc treatment during the first 3 days after SC; G-CSF promoted the recovery of long-term motor function and the protection of neurons following SCI.

### Zinc Inhibits Apoptosis After SCI *via* G-CSF

**Figure 7:**
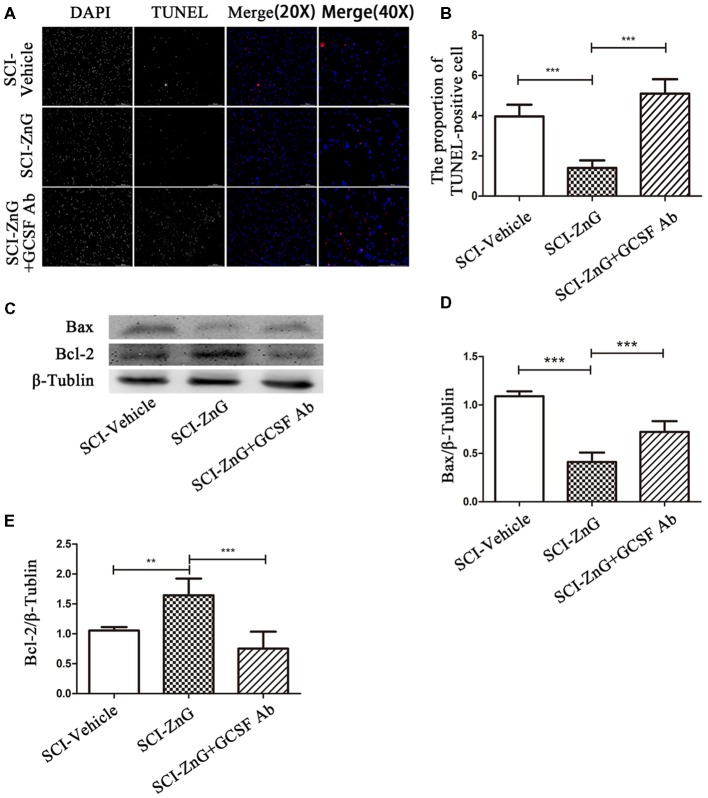
Zinc treatment reduced apoptosis in spinal cord tissue by G-CSF after SCI. **(A)** Transferase UTP Nick End Labeling (TUNEL)/4′,6-diamidino-2-phenylindole (DAPI) labeling was used to count the number of TUNEL-positive cell in the SCI-Vehicle, SCI-ZnG and SCI-ZnG+GCSF Ab groups. **(B)** Quantification analysis of the proportion of TUNEL-positive cells in each group (*n* = 6 per group, one-way ANOVA followed by Bonferroni’s *post hoc* test). **(C)** Representative western blots for Bax and Bcl-2 expression, and the loading control (β-Tublin), in SCI-Vehicle, SCI-ZnG and SCI-ZnG+GCSF Ab groups at 3 days post-injury. **(D,E)** Quantification analysis of the expression levels of Bax and Bcl-2 (*n* = 6 per group, one-way ANOVA followed by Bonferroni’s *post hoc* test). Data are expressed as means ± SD, **p < 0.01, ***p < 0.001.

Apoptosis occurs following SCI as a result of the down-regulation of anti-apoptotic Bcl-2 and the up-regulation of pro-apoptotic Bax (Emery et al., 1998). We observed that on the third day after SCI, the level of Bax was lower in the SCI-ZnG group than in vehicle group (*P* < 0.001); the level of Bcl-2 was higher in the SCI-ZnG group than in the vehicle group (*P* < 0.001). However, these phenomena were reversed after the administration of G-CSF neutralizing antibody (Figures 7C–E). Furthermore, we performed TUNEL/DAPI staining (Figure 7A). As shown in Figure 7B, the proportion of TUNEL-positive cells were significantly lower in the SCI-ZnG group than that in the vehicle group and the anti-GCSF group. These findings confirmed that zinc treatment inhibited apoptosis after SCI, and that the inhibition of apoptosis was mainly mediated by G-CSF.

### Zinc Inhibits Neuronal Apoptosis After SCI *via* G-CSF

**Figure 8:**
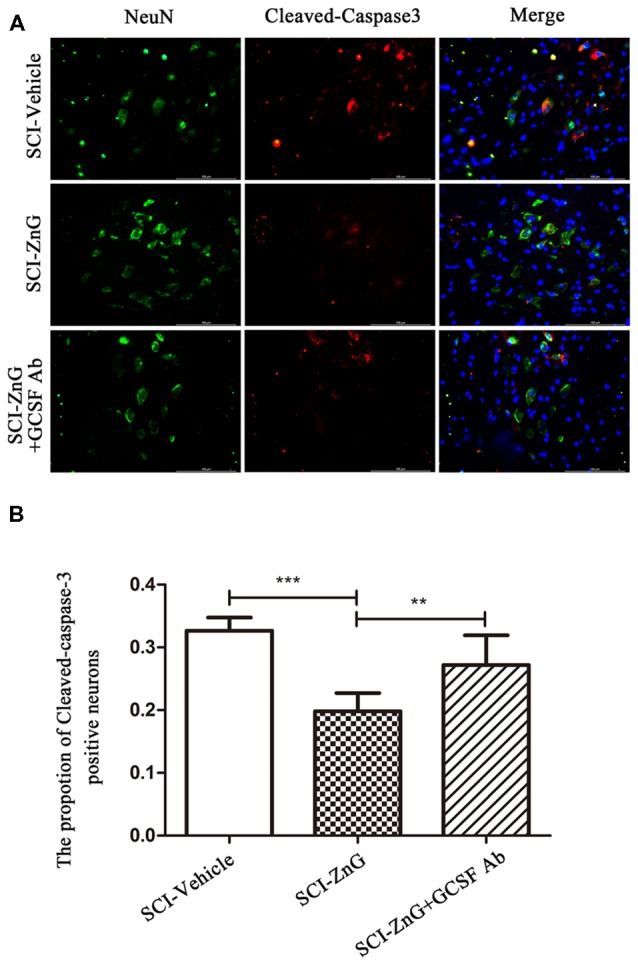
Zinc treatment reduced neuronal apoptosis by G-CSF after SCI. **(A)** Representative double immunofluorescent staining for Cleaved-caspase-3-positive cells (red), NeuN (green), and DAPI (blue) in SCI of SCI-Vehicle, SCI-ZnG and SCI-ZnG+GCSF Ab mice at 21 days post-injury.** (B)** Quantification of Cleaved-caspase-3-positive cells co-labeled with NeuN to reveal apoptotic neurons (*n* = 6–7 per group, one-way ANOVA followed by Bonferroni’s *post hoc* test). Data are expressed as means ± SD, **p < 0.01, ***p < 0.001.

To further assess the effects of zinc and G-CSF on neuronal apoptosis, we performed double immunofluorescent staining for Cleaved-caspase-3 and NeuN (Figures 8A,B). The immunoreactivity of Cleaved-caspase3-positive neurons in the SCI-ZnG group was lower than the SCI-Vehicle group (SCI-ZnG vs. SCI-Vehicle, *P* < 0.001) and the SCI-ZnG+GCSF Ab group (SCI-ZnG vs. SCI-ZnG+GCSF Ab, *P* < 0.01). These results suggest that short-term zinc therapy can benefit the survival of neurons after SCI, and achieves this affect by promoting the expression of G-CSF and reducing the incidence of apoptosis.

## Discussion

In the present study, we examined the role of zinc in regulating G-CSF secretion and neuronal protection after SCI. The pathological process of SCI includes primary injury and secondary injury. The primary injury is a local injury caused by external force acting directly upon the spinal cord, and is characterized by bleeding, edema and compression. Secondary injury is mainly caused by the release and interaction of cytokines, resulting in the further death of neurons and glial cells, and the extension of injury to higher segments. Such damage is mainly caused by the cascade of cytokines after SCI, which leads to permanent functional defects (Kwon et al., 2004). Previous research by our team found that zinc supplementation facilitated the treatment of (Wang et al., 2011b). On the basis of our previous study, the present aim was to further explore the possible mechanism of zinc protection against SCI. We hypothesized that studying the changes of cytokines induced by zinc after SCI would help us to understand how zinc exerts neuroprotective effects.

Both high and low concentrations of zinc are known to cause cell death in nerve cells (Morris and Levenson, 2013; Seth et al., 2015). Adamo reported that low levels of zinc induces apoptosis in IMR-32 neuroblastoma cells and primary cortical neurons (Adamo et al., 2010). Zinc is one of the trace elements, and a high concentration of zinc will lead to cell apoptosis. In this experiment, we injected different concentrations of ZnG (15, 30, 60, 120 mg/kg) intraperitoneally and found that a concentration of 30 mg/kg was the maximum dose with which to maintain survival rate of mice on day 3 at 100%. These results suggest that intraperitoneal injection of a high concentration of zinc is not conducive to the recovery of SCI. furthermore, in a follow-up experiment, we found that 30 mg/kg of ZnG was more beneficial to the recovery of SCI than a vehicle group. This result is consistent with the high and low zinc levels described in other literature, which are not conducive to the survival of neurons (Hsieh et al., 2015; Seth et al., 2015).

To investigate the effect of zinc on cytokines during the first 3 days after SCI, we used a cytokine antibody array C2000 as a research method with which to detect the effect of zinc on the changes of local cytokines at 1 and 3 days after SCI. Results suggested that the expression of G-CSF protein during the first 3 days after zinc treatment increased continuously; mRNA expression followed the same trend. We believe that G-CSF plays an important role in the neuroprotective effect of zinc. The expression of G-CSF was the highest of all cytokines on day 1 after SCI. Thus, we consider that an increase in G-CSF might be one of the initial changes in cytokine levels mediated by zinc after SCI, and that this change may be related to changes in cytokine profile on day 3 after SCI. In order to detect changes in cytokines on the third day, we took samples of spinal cord tissue from above and below the injury point 24 h after the administration of zinc and G-CSF neutralizing antibody after SCI in mice; changes in cytokine profile were then detected by qRT-PCR. The results suggested that G-CSF neutralization significantly increased the levels of inflammatory cytokines (TNF-α, IL-6) and decreased some chemokines (e.g., the CXCL and CCL families). Given these, we speculate that G-CSF changes first after SCI treatment with zinc, and that increased levels of G-CSF increases the transcription of chemokines and reduces the transcription of inflammatory factors over the subsequent course of disease. This hypothesis forms the basis or our future research directive.

In the present research, we speculate that G-CSF is able to alter the transcription of other cytokines and consider that G-CSF changes the mRNA expression of other cytokines *via* nuclear transcription factors. We investigated changes in cytokine profile on day 3 after SCI by reviewing the relevant literature. We found that NF-κB is involved in the expression of a range of cytokines, including KC, VEGF, E-selectin, TPO, IL-6, G-CSF and CD40 (Kiriakidis et al., 2003; Nazar et al., 2012; Cao et al., 2014; Huang et al., 2014; Goel et al., 2015; Yang et al., 2017). Therefore, we suspect that G-CSF changes the expression of downstream cytokines *via* the NF-κB signaling pathway. However, it is interesting to note that the upstream and downstream relationships between G-CSF and NF-κB are somewhat different. This discrepancy may be due to the positive feedback correlation between G-CSF and NF-κB. Based on this line of thinking, we hypothesize that zinc increases the expression of G-CSF *via* the NF-κB signaling pathway, and that increased levels of G-CSF increases its own expression *via* positive feedback from the NF-κB signaling pathway, and that changes in the transcription and expression of other cytokines downstream occur through the NF-κB signaling pathway. Besides, we also observed an interesting phenomenon in the RAW264.7 cell line, in that the expression of G-CSF protein after PDTC treatment was close to zero. We speculate that NF-κB might represent the most important signal pathway for G-CSF production by M/Ms.

G-CSF plays different roles in different tissues of the human body. G-CSF can increase the expression of granulocytes in peripheral blood to treat leukopenia, but in the central nervous system, G-CSF can protect neurons and promote the regeneration of vascular endothelial cells. Generally speaking, at present, most people believe that G-CSF is mainly expressed and secreted by neurons in the central nervous system (Schneider et al., 2005), and other cells also participate in the secretion of even a very small amount of G-CSF. In our experiments, we found that the expression of G-CSF continuously increased in mice over the course of 3 days after SCI mice were treated with zinc, and that this increase was not caused by large scale secretion from neurons, but mainly by microglia. We believe that this difference is due to certain characteristics of the damage environment after SCI. Neurons within the injured area would have received a huge blow, and the number of M/Ms in this damaged area thus increases because of local inflammation by inflammatory cells recruited by the peripheral blood. As a result, the observed increase in G-CSF comes mainly from M/Ms. G-CSF also has an important non-hematopoietic function in the central nervous system, and G-CSF Receptor (G-CSFR) is the only site of action for G-CSF. G-CSFR is expressed not only in neutrophils, monocytes, platelets and other cells, but also in endothelial cells, neurons and glial cells in non-hematopoietic cells. Therefore, when G-CSF acts on the G-CSFR of neurons, it will have a certain protective effect upon injured neurons. Recent studies have shown that VEGF, E-selectin and TPO have a protective effect on ischemic and anoxic nerve tissue (Bernstein et al., 1986; Yun et al., 2008; Emerich et al., 2010). Therefore, we believe that G-CSF not only plays a neuroprotective role by acting on G-CSFR, but also through G-CSFRs expressed on other cell types in the central nervous system, thus promoting the expression of cytokines such as VEGF, E-selectin and TPO, and thus exerting neuroprotective effects.

It should be noted that there are some limitations in this study which need to be considered. First, after the intraperitoneal injection of ZnG, the concentration of local Zn^2+^ remained unknown. We did not determine the concentration of local Zn^2+^ at the injury site. Second, the effects of G-CSF on other central nervous system cells and other cytokines are still unclear. We will investigate such factors in our future research. It will also be necessary to identify the local concentration of Zn^2+^ and clarify other mechanisms of neuroprotective effect of G-CSF.

## Conclusion

Zinc treatment increases the expression of G-CSF secreted by M/Ms, which leads to reduced neuronal apoptosis after SCI.

## Data Availability

All datasets generated for this study are included in the manuscript and the supplementary files.

## Author Contributions

XL, SC and LM wrote the main manuscript. DL and CX collected samples. XL and LM analyzed data, discussed results and prepared figures. XL, LM, HT and XM designed the experiment. LM and XM revised the manuscript.

## Conflict of Interest Statement

The authors declare that the research was conducted in the absence of any commercial or financial relationships that could be construed as a potential conflict of interest.

## Supplementary Material

FIGURE S1 The schematic diagram of cytokine antibody array. **(A)** Three films of C3/C4/C5 form a piece of cytokine antibody array. Each film is placed in a hole, each cytokine antibody array needs three holes. **(B)** Red stands for positive control, green for negative control, and each protein consists of two adjacent points. The proteins on each film are shown in the figure **(C)**.

## Abbreviations

SCI: Spinal cord injury
G-CSF: Granulocyte Colony Stimulating Factor
ZnG: Zinc Gluconate
BMS: Basso Mouse Scale
M/Ms: Microglia/macrophages
NF-κB: Nuclear factor-kappa B
qRT-PCR: Quantitative real-time PCR analysis
RPS18: Ribosomal protein S18
DAPI: 4′,6-diamidino-2-phenylindole
MTS: CellTiter 96^®^ AQueous One Solution Cell Proliferation Assay
PDTC: Pyrrolidinedithiocarbamate
TNF-α: Tumor necrosis factor alpha
IL-6: Interleukin 6
CXCL: Chemokine (C-X-C motif) ligand
CCL: (C-C motif) ligand
KC: Chemokine (C-X-C motif) ligand 1
VEGF: Vascular endothelial growth factor
E-Selectin: CD62 antigen-like family member E
TPO: Thyroid peroxidase
G-CSFR: Granulocyte Colony Stimulating Factor Receptor.
