# Supplementary figures and images for: Zinc Improves Functional Recovery by Regulating the Secretion of Granulocyte Colony Stimulating Factor From Microglia/Macrophages After Spinal Cord Injury
Source: Front Mol Neurosci. 2019 Feb 1;12:18. doi: 10.3389/fnmol.2019.00018 (PMC6367229; doi:10.3389/fnmol.2019.00018)

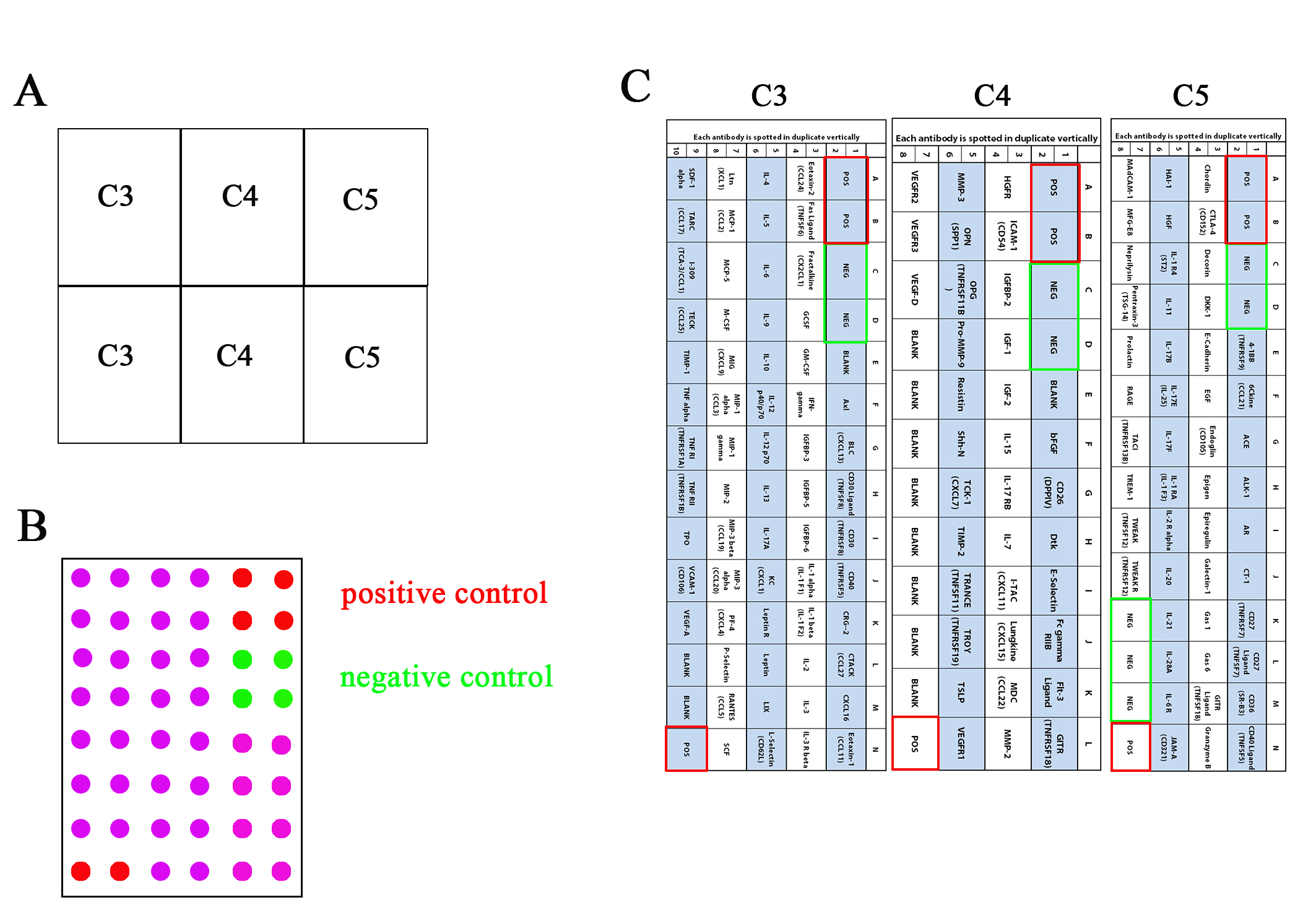

Supplement: FIGURE S1 — The schematic diagram of cytokine antibody array. (A) Three films of C3/C4/C5 form a piece of cytokine antibody array. Each film is placed in a hole, each cytokine antibody array needs three holes. (B) Red stands for positive control, green for negative control, and each protein consists of two adjacent points. The proteins on each film are shown in the figure (C). [file Image_1.TIF]
